# Novel Hybrid Peptide Cathelicidin 2 (1-13)-Thymopentin (TP5) and Its Derived Peptides with Effective Antibacterial, Antibiofilm, and Anti-Adhesion Activities

**DOI:** 10.3390/ijms222111681

**Published:** 2021-10-28

**Authors:** He-Nan Guo, Yu-Cui Tong, Hui-Li Wang, Jing Zhang, Zhong-Xuan Li, Zaheer Abbas, Tian-Tian Yang, Meng-Yao Liu, Pei-Yao Chen, Zheng-Chang Hua, Xiao-Na Yan, Qiang Cheng, Marhaba Ahmat, Jun-Yong Wang, Lu-Lu Zhang, Xu-Biao Wei, Xiu-Dong Liao, Ri-Jun Zhang

**Affiliations:** 1 Laboratory of Feed Biotechnology, State Key Laboratory of Animal Nutrition, College of Animal Science and Technology, China Agricultural University, Beijing 100193, China; ghn_657@cau.edu.cn (H.-N.G.); 15956910334@163.com (Y.-C.T.); wanghlzn@126.com (H.-L.W.); zhangjing123@cau.edu.cn (J.Z.); Zaheerabbas113@yahoo.com (Z.A.); ytt2020@cau.edu.cn (T.-T.Y.); 17852027051@163.com (M.-Y.L.); y18983829640@163.com (P.-Y.C.); 13121125788@163.com (Z.-C.H.); chengqiangcool@163.com (Q.C.); malika511@126.com (M.A.); wangjy9722@163.com (J.-Y.W.); 2 College of Bioengineering, Sichuan University of Science & Engineering, Zigong 643000, China; Lee_zx20@yeah.net; 3 College of Animal Science and Technology, Hebei Normal University of Science & Technology, Qinhuangdao 066004, China; y98301219@126.com; 4 School of Pharmaceutical Sciences, Tsinghua University, Beijing 100084, China; zhanglulu150327@126.com (L.-L.Z.); weixubiao01@126.com (X.-B.W.); 5 Tsinghua-Peking Center for Life Sciences, Tsinghua University, Beijing 100084, China; 6 Mineral Nutrition Research Division, State Key Laboratory of Animal Nutrition, Institute of Animal Sciences, Chinese Academy of Agricultural Sciences, Beijing 100193, China; liaoxd56@163.com

**Keywords:** antimicrobial peptides, cathelicidin 2 (1-13)-thymopentin (TP5), multidrug resistance, *Staphylococcus aureus*, antibacterial, antibiofilm, anti-adhesion

## Abstract

The increasing numbers of infections caused by multidrug-resistant (MDR) pathogens highlight the urgent need for new alternatives to conventional antibiotics. Antimicrobial peptides have the potential to be promising alternatives to antibiotics because of their effective bactericidal activity and highly selective toxicity. The present study was conducted to investigate the antibacterial, antibiofilm, and anti-adhesion activities of different CTP peptides (CTP: the original hybrid peptide cathelicidin 2 (1-13)-thymopentin (TP5); CTP-NH_2_: C-terminal amidated derivative of cathelicidin 2 (1-13)-TP5; CTPQ: glutamine added at the C-terminus of cathelicidin 2 (1-13)-TP5) by determining the minimal inhibitory concentrations (MICs), minimal bactericidal concentrations (MBCs), propidium iodide uptake, and analysis by scanning electron microscopy, transmission electron microscopy, and confocal laser scanning microscopy). The results showed that CTPs had broad-spectrum antibacterial activity against different gram-positive and gram-negative bacteria, with MICs against the tested strains varying from 2 to 64 μg/mL. CTPs at the MBC (2 × MIC 64 μg/mL) showed strong bactericidal effects on a standard methicillin-resistant *Staphylococcus aureus* strain ATCC 43300 after co-incubation for 6 h through disruption of the bacterial membrane. In addition, CTPs at 2 × MIC also displayed effective inhibition activity of several *S. aureus* strains with a 40–90% decrease in biofilm formation by killing the bacteria embedded in the biofilms. CTPs had low cytotoxicity on the intestinal porcine epithelial cell line (IPEC-J2) and could significantly decrease the rate of adhesion of *S. aureus* ATCC 43300 on IPEC-J2 cells. The current study proved that CTPs have effective antibacterial, antibiofilm, and anti-adhesion activities. Overall, this study contributes to our understanding of the possible antibacterial and antibiofilm mechanisms of CTPs, which might be an effective anti-MDR drug candidate.

## 1. Introduction

Although antibiotics were once considered the most effective anti-infective drugs, the abuse of antibiotics has provoked the development of drug-resistant (DR) and multidrug-resistant (MDR) pathogens. Among such pathogens, *Staphylococcus aureus* is a gram-positive pathogen that causes biofilm-associated infections, including pulmonary, urinary, and skin infections [1]. Biofilms constitute a basic survival strategy of bacteria in hostile environments [2], in which the bacteria are embedded in an extracellular matrix [3]. Moreover, biofilm formation depends on the regulation of the quorum sensing (QS) system [4], which is connected to intercellular communication in bacteria, making them sense the population threshold and express various virulence factors [5]. During a pathogen infection, biofilms can impede the normal function of the host immune system and antibiotic therapy [6,7,8,9]. During the course of the disease, the adhesion of pathogens to epithelial cells is an essential first step for the pathogens to survive and colonize the gastrointestinal tract [10]. Therefore, it is important to find safe and effective antibiotic substitutes with antibacterial, antibiofilm, and anti-adhesion activities. Among a variety of candidates, antimicrobial peptides (AMPs), with positively charged and hydrophobic residues, have the potential to be promising alternatives to antibiotics [11,12].

The antibacterial mechanisms of AMPs mainly include membrane destruction and non-membrane destruction. The membrane-targeting mechanisms mainly rely on the electrostatic adsorption and hydrophobicity of the AMPs to disrupt the cell membrane, resulting in the outflow of the cell contents followed by lysis and death of the bacteria [13,14]. However, non-membrane destruction mechanisms work mainly through the inhibition of protein biosynthesis, including the depressed transcription or translation of key proteins [13,15,16,17]. AMPs have also been reported to arrest cell division in intracellular environments [18,19]. Though biofilms originate from planktonic bacteria, they differ in their properties. Therefore, the antibiofilm mechanisms of AMPs include the following aspects: direct damage to the bacterial cell membrane [20,21,22]; interference of the QS system [23,24,25]; reduction of extracellular polymeric substances (EPS) accumulation [26]; and inhibition of stringent response [27].

We previously designed and screened a hybrid peptide CTP (RWGRFLRKIRRFRRKDVT), which is composed of the active center of cathelicidin 2 (CATH2) and thymopentin (TP5) (CTP, CATH2 (1-13)-TP5) and exhibits anti-inflammatory and immune activities [28]. We found that the C-terminal amidated CTP (CTP-NH_2_) had lower cytotoxicity in mouse macrophage (RAW264.7) cells and better anti-inflammatory effects in lipopolysaccharide (LPS)-induced inflammation in vitro and In vivo [28]. However, the amidation reaction of the C-terminal of CTP-NH_2_ is difficult to achieve directly in a heterologous expression. The addition of a residue containing an amide group, such as glutamine or asparagine, to the C-terminal of AMPs has been reported to significantly improve its bactericidal activity [29,30].

In this study, we hypothesized that CTP and its derivative peptides may exert strong antibacterial, antibiofilm, and anti-adhesion activities against *S. aureus*. We introduced glutamine (Q) at the C-terminal of the peptide CTP to generate CTPQ. The present study was aimed at evaluating the antibacterial, antibiofilm of CTPs, including CTP, CTP-NH_2_, and CTPQ. To evaluate the anti-adhesion activity of CTPs against *S. aureus* on mammal intestinal epithelial cells, we established an *S. aureus*-adhered model with an intestinal porcine epithelial (IPEC-J2) cell in vitro.

In the previous studies related to an antimicrobial peptide, they mainly focused on the antibacterial, antibiofilm or anti-adhesion effects against gram-negative pathogens, such as enterohaemorrhagic *Escherichia coli* (EHEC) and enterotoxigenic *E. coli* (ETEC) [31,32,33,34], while lacking attention to gram-positive bacteria, especially methicillin-resistant *Staphylococcus aureus* (MRSA) that can form biofilms. Herein, we firstly explored the antibacterial spectrum of our anti-inflammatory peptides CTPs, and we mainly paid attention to the antibacterial, antibiofilm, and anti-adhesion activities of CTPs against an MRSA target strain *S. aureus* ATCC 43300. In general, this study was aimed to enrich the knowledge on bioactivities and mechanisms of CTPs, and to provide a theoretical basis for the efficient expression of highly active hybrid peptide CTPs.

## 2. Results

### 2.1. Physiochemical Properties of Peptides

**Figure 1:**
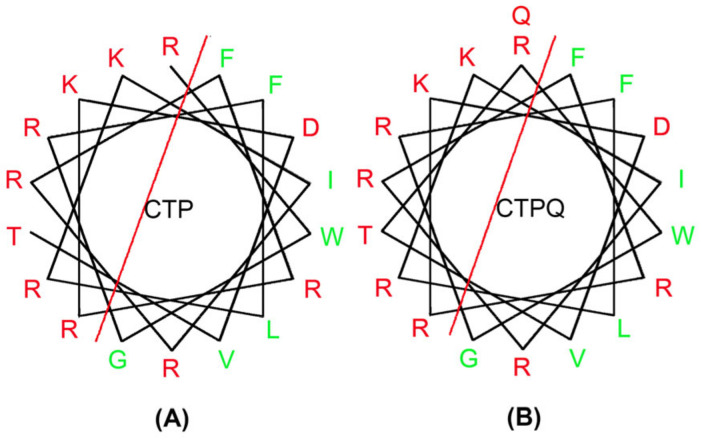
Helical wheel diagram of peptides (**A**) CTP (cathelicidin 2 (CATH2) and thymopentin (TP5)) and (**B**) CTPQ (addition of glutamine at the C-terminal of cathelicidin 2 (CATH2) and thymopentin (TP5)) using a website (https://www.bioinformatics.nl/cgi-bin/emboss/pepwheel (accessed on 12 February 2021)). The hydrophobic residues were shown in green, and the hydrophilic residues were shown in red. In addition, the red line separated the hydrophilic and hydrophobic residues.

As shown in Table 1, the addition of glutamine did not affect the net charge number of the CTP. However, there was a slight decrease in the hydrophobic moment, an important index to evaluate the amphiphilicity, which was consistent with the increase in hydrophobicity caused by glutamine. The helical wheel diagram can predict the hydrophobic residue distribution of the peptides. Here, the introduction of a glutamine at the C-terminal of the CTP affected its amphiphilicity by increasing the number of hydrophobic residues (Figure 1).

### 2.2. Determination of Minimal Inhibitory Concentrations (MICs) and Minimal Bactericidal Concentrations (MBCs)

The MICs of CTPs (CTP, CTP-NH_2_, and CTPQ) against all of the tested strains including *S*. *aureus*, *Shigella castellani*, *Salmonella typhimurium*, *Salmonella pullorum*, *Escherichia coli*, and *Pseudomonas aeruginosa* did not exceed 64 μg/mL, but the MBCs varied from 4 to 512 μg/mL (Table 2). CTP-NH_2_ showed the best comprehensive antibacterial performance against all of the treated strains in comparison with CTP and CTPQ. Moreover, the lowest MICs (CTP 4 μg/mL, CTP-NH_2_ 2 μg/mL, and CTPQ 4 μg/mL) and MBCs (CTP 4 μg/mL, CTP-NH_2_ 8 μg/mL, and CTPQ 4 μg/mL) were observed against *S. aureus* CVCC 1882, compared to the other treated strains. CTPs also showed effective antibacterial activity against *S. aureus* ATCC 43300, a standard MRSA strain, with an MIC of 32 μg/mL and an MBC of 64 μg/mL.

Although CTP and CTPQ shared consistently similar MICs against almost all of the tested strains, the MICs of CTP against *P. aeruginosa* ATCC 27,853 and *P. aeruginosa* CGMCC 1.10712 were higher than those of CTPQ.

### 2.3. Inhibited Zone Assay

**Figure 2:**
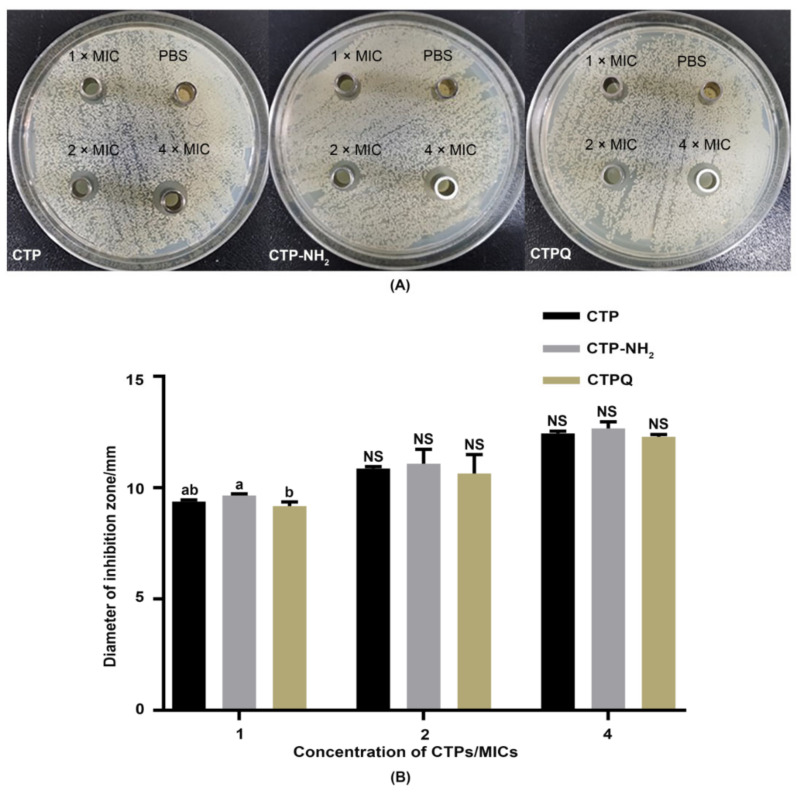
(**A**) The inhibition zone of *S. aureus* ATCC 43300 treated with CTPs at 1, 2, and 4 × MIC (32, 64 and 128 μg/mL) and (**B**) their diameters. Data were given as the mean value ± SD from three biological replicates. ^a,b^: Different lowercase letters mean significantly different (*p* < 0.05) values were expressed as mean ± SEM; NS: not significant (*p* > 0.05) (*n* = 3).

To visually evaluate the antibacterial effects of CTPs, an inhibition zone assay of *S. aureus* ATCC 43300 by CTPs was conducted using the Oxford cup method. As shown in Figure 2, the inhibition zone of CTP-NH_2_ was significantly larger (*p* < 0.05) than that of CTP and CTPQ at 1× MIC. However, were no significant differences (*p* > 0.05) among the inhibition zones of the three peptides at 2 or 4 × MIC. Overall, the sizes of the CTP-inhibited zones increased by approximately 3 mm when the concentration of the peptides increased from 1 × MIC to 4 × MIC.

### 2.4. In Vitro Time-Kill Curve Assay

**Figure 3:**
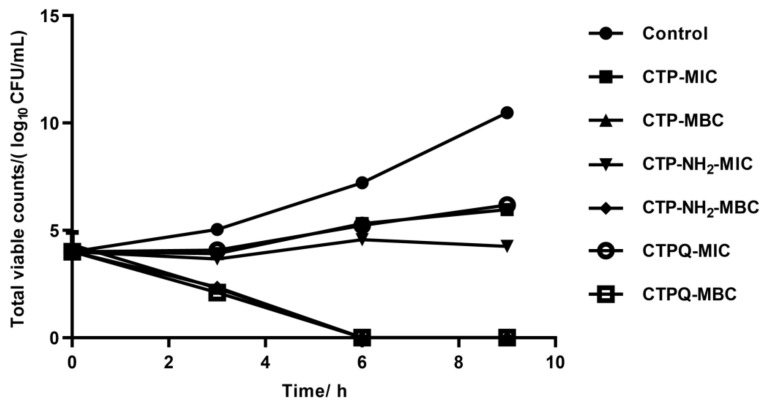
Time-kill curves of *S. aureus* ATCC 43300 treated with CTPs (CTP, CTP-NH_2_, and CTPQ) at the MIC (32 μg/mL) and 2 × MIC (64 μg/mL). Data were given as the mean value ± SD from three biological replicates.

**Figure 4:**
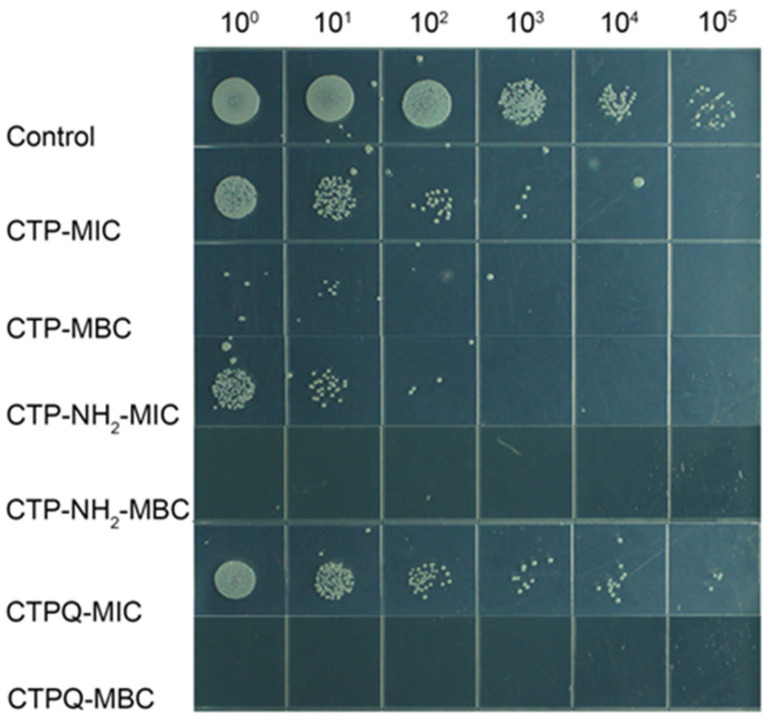
Spot plates of *S. aureus* ATCC 43300 treated with CTPs (CTP, CTP-NH2, and CTPQ) treatment at the MIC (32 μg/mL) and the MBC (64 μg/mL) for 6 h. 10^0^–10^5^: dilution ratio of bacteria suspension.

The time-kill kinetics of CTPs against *S. aureus* ATCC 43300 were further investigated by counting the viable cells upon CTP treatment at the MIC and 2 × MIC (MBC). As shown in Figure 3, the bacteria were completely killed upon exposure to the peptides for 6 h at 2 × MIC. The killing efficiencies of *S. aureus* by CTPs declined at the MIC. Specifically, all of the CTPs were able inhibit the growth of pathogenic bacteria at the MIC in the initial 3 h. Thereafter, CTP-NH_2_ was still able to exert a fairly strong antibacterial effect till 9 h, while that of CTP and CTPQ decreased to some extent. These results indicate that although all three peptides have identical MICs and MBCs against *S. aureus* ATCC 43300, CTP-NH_2_ exhibits a better bactericidal property than the other two peptides. We also performed a spot plate assay (Figure 4), and the results indicated that the trend of viable colony reduction was similar to the time-kill curves.

**Figure 5:**
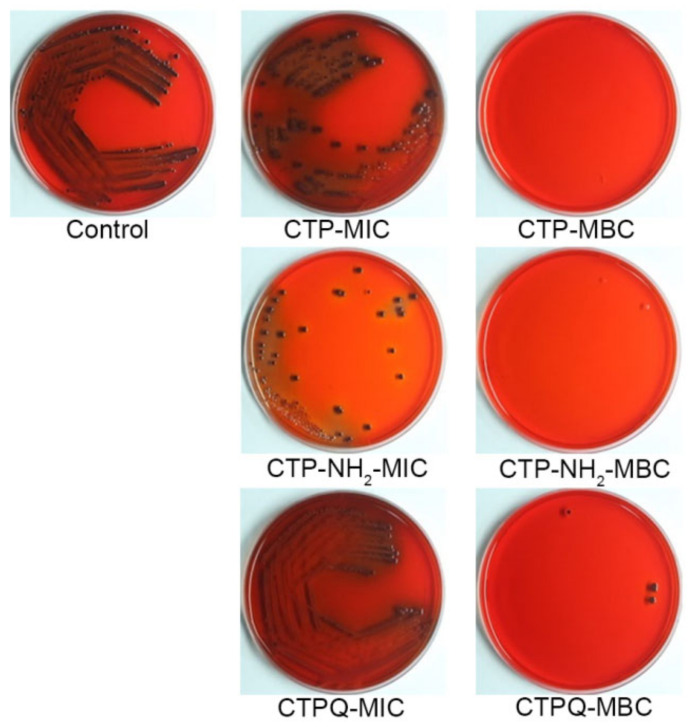
CRA plate of *S. aureus* ATCC 43300 treated with CTPs (CTP, CTP-NH_2_, and CTPQ) treatment at the MIC (32 μg/mL) and the MBC (64 μg/mL) for 9 h.

The *S. aureus* colony color and morphology on Congo red agar (CRA) plates could reveal whether slime is produced, which is connected to the formation of biofilms [35]; in short, black colonies of *S. aureus* with a dry crystalline consistency indicate the production of slime and a positive result. In contrast, red/smooth colonies represent a negative result and no slime production [35]. When treated with CTPs at the MIC, the slime generation by the colonies decreased (Figure 5). In particular, colonies treated with CTP-NH_2_ and CTPQ at 1 × MIC showed a lighter color than the control, demonstrating that slime production was inhibited. However, when treated with the CTPs at the MBC after co-culture for 9 h, almost no colony remained due to the bactericidal effect. These results showed that CTPs might inhibit biofilm formation. Therefore, the antibiofilm activity of CTPs was further evaluated in this study.

### 2.5. Cell Membrane Integrity Evaluation and Morphological Observation of S. aureus 

**Figure 6:**
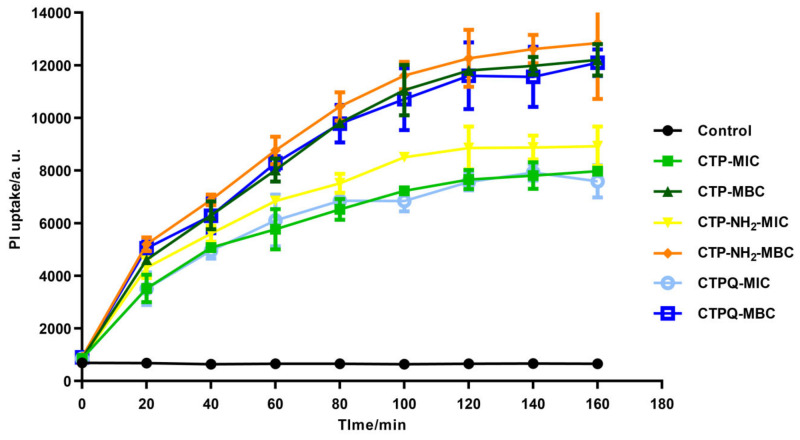
PI uptake of *S. aureus* ATCC 43300 treated with CTPs (CTP, CTP-NH2, and CTPQ) treatment at the MIC (32 μg/mL) and the MBC (64 μg/mL). Results are expressed in arbitrary units (a. u.).

The bactericidal mechanism of CTPs was investigated using a propidium iodide (PI) uptake assay, in which PI binds to intracellular nucleic acids of membrane-broken cells, thus producing red fluorescence. The PI fluorescence intensity indicates the severity of damage to the cell membrane integrity. As seen in Figure 6, all of the CTPs were able to increase the uptake of PI at the MIC and the MBC, demonstrating the disruption of the *S. aureus* cell membrane by CTPs. Moreover, a higher PI uptake was induced by CTPs at the MBC (2 × MIC) than at the MIC. Meanwhile, CTP-NH_2_ induced a better PI uptake than the other two peptides at either the MIC or the MBC, which was consistent with the results of the time-kill curves.

**Figure 7:**
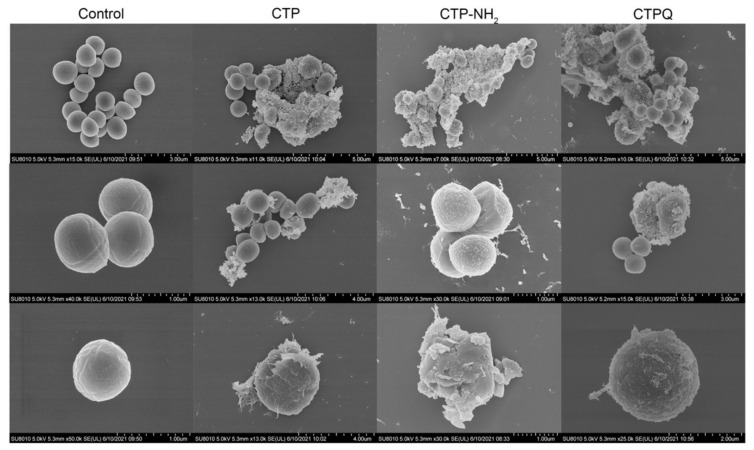
SEM assay of *S. aureus* ATCC 43300 treated with CTPs (CTP, CTP-NH_2_, and CTPQ) at the MIC (32 μg/mL).

Furthermore, changes in the *S. aureus* ATCC 43300 cell surface induced by CTPs at the MBC were examined by scanning electron microscopy (SEM) (Figure 7). The surface of the cell membrane in the control group was intact and smooth, and most of the cells were observed in different states of division. In contrast, their counterparts in the CTP-treated groups became rough and swollen, losing their original morphology. In addition, cell aggregation and adhesion caused by intracellular matrix leakage were also observed in the majority of the broken cells. In particular, CTP-NH_2_-treated samples contained the total least number of complete cells, confirming that this peptide had the best bactericidal effect. These results proved again that the bactericidal mechanism of CTPs was based on the destruction of the bacterial cell membrane.

**Figure 8:**
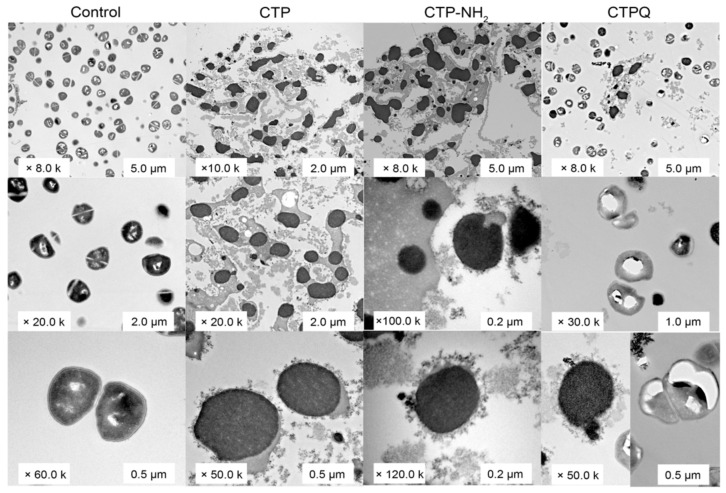
TEM assay of *S. aureus* ATCC 43300 treated with CTPs (CTP, CTP-NH2, and CTPQ) at the MIC (32 μg/mL).

The intracellular structure of *S. aureus* treated with CTPs was further illustrated by transmission electron microscopy (TEM) (Figure 8). The structures of the cells in the control group were intact and plump, rounded with hierarchical clear cell walls, cell membranes, and protoplasts. In addition, the division of the control cells was normal with clear constrictions. Conversely, *S. aureus* treated with CTPs at the MIC showed cellular damage, which mainly included DNA aggregation and ribosome cohesion (light and dark areas, respectively), blurred or even disappeared boundaries of the cell walls or membranes, irregular cell shape, cytoplasmic solidification, formation of intracellular vacuoles, and cell lysis and extravasation of the contents. Thus, it was obvious that CTPs could also effectively disrupt the intracellular structures of *S. aureus* after membrane destruction.

### 2.6. Antibiofilm Activity Assay

Biofilm formation could improve the pathogenicity of *S. aureus* by increasing the synthesis of the virulence factors, resulting in drug resistance in the strains. To explore whether CTPs had an antibiofilm activity against several strains of *P. aeruginosa* (gram-negative strain) and *S. aureus* (gram-positive strain), we conducted a preliminary investigation using crystal violet staining.

**Figure 9:**
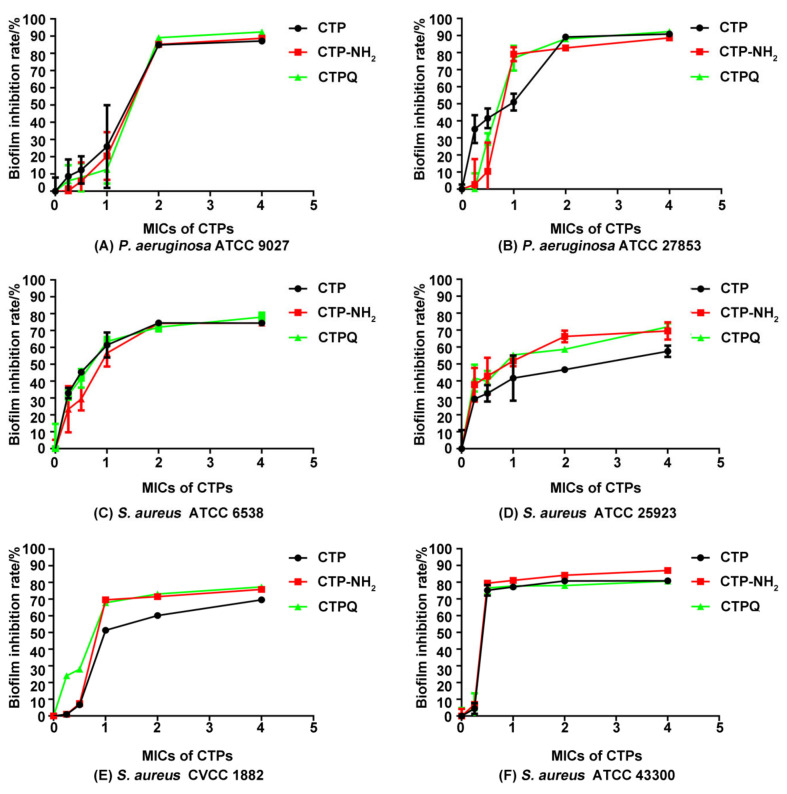
Antibiofilm activity of CTPs (CTP, CTP-NH2, and CTPQ) against (**A**) *P. aeruginosa* ATCC 9027, (**B**) *P. aeruginosa* ATCC 27853, (**C**) *S. aureus* ATCC 6538, (**D**) *S. aureus* ATCC 25923, (**E**) *S. aureus* CVCC 1882, and (**F**) *S. aureus* ATCC 43300.

**Figure 10:**
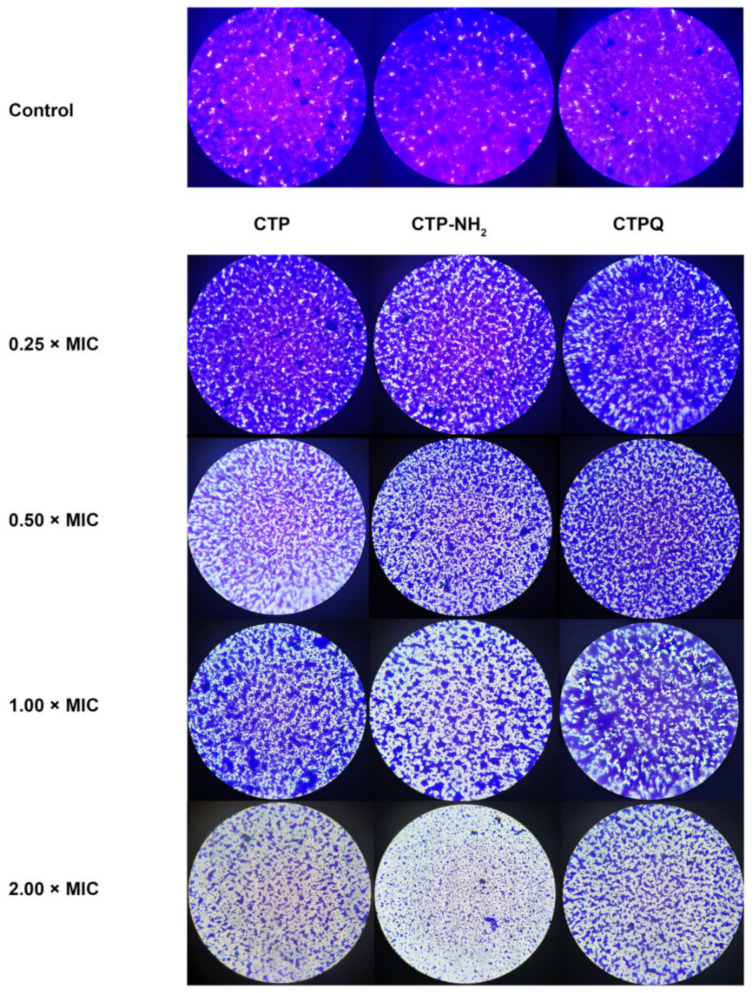
Optical microscope observation of *S. aureus* ATCC 43300 biofilm treated by CTPs (CTP, CTP-NH_2_, and CTPQ).

The results showed that all of the CTPs exhibit an efficient antibiofilm activity against all of the tested strains in a dose-dependent manner (Figure 9). Interestingly, there were differences in their antibiofilm effects on different strains. There was no difference in biofilm inhibition rates of CTPs (≥1 × MIC) against *P. aeruginosa* ATCC 27853, *P. aeruginosa* ATCC 9027, *S. aureus* ATCC 43300, and *S. aureus* ATCC 6538. However, for *S. aureus* ATCC 25923 and *S. aureus* CVCC 1882, the antibiofilm activity of CTP was weaker than that of CTP-NH_2_ and CTPQ, which might be due to the specificity of the cell membrane composition, resulting in different combinations and destruction abilities of the peptides. In particular, both CTP-NH_2_ and CTPQ inhibited biofilm formation of all of the tested strains at sub-lethal concentrations (MIC), and showed 50–90% biofilm inhibition rates at 2 and 4 × MIC. Furthermore, the antibiofilm effects of CTPs against *S. aureus* ATCC 43300 were also observed after crystal violet staining using an optical microscope (Figure 10). Among the three peptides, CTP-NH_2_ had the best inhibitory effect on biofilm formation by *S. aureus* ATCC 43300.

### 2.7. Confocal Laser Scanning Microscope (CLSM) Assay and Quantitative Real-Time Polymerase Chain Reaction (qRT-PCR) Results of Biofilm-Associated Genes

**Figure 11:**
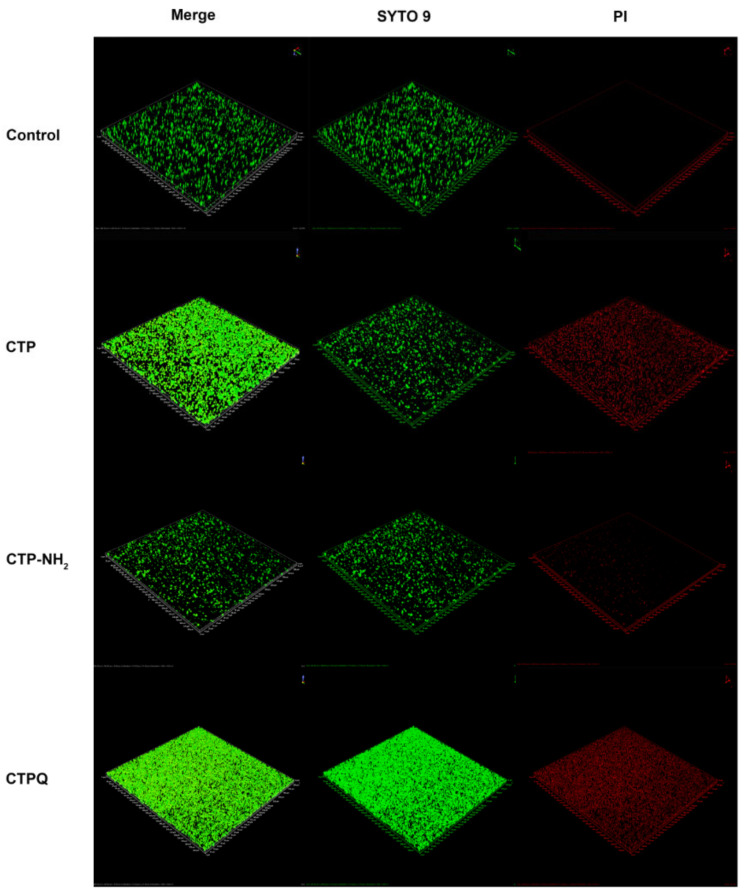
CLSM assay of *S. aureus* ATCC 43300 biofilm treated by CTPs (CTP, CTP-NH_2_, and CTPQ) at the MBC (64 μg/mL) with 40 × oil lens.

Since crystal violet staining of biofilms does not distinguish between live and dead cells, we used SYTO 9/PI staining to mark the live/dead cells embedded in the biofilm of *S. aureus* ATCC 43300 by CLSM. SYTO 9 can penetrate the cell membrane of all live/dead cells, while PI can only enter dead cells and bind to nucleic acids. As shown, all of the CTPs at the MBC could destroy the membrane of *S. aureus* to inhibit the formation of biofilms, where broken cells were labeled with red fluorescence by PI (Figure 11). Dead cells in all of the CTP-treated biofilms increased after peptide treatment. These results indicated that all CTPs could inhibit biofilm formation by *S. aureus* via membrane destruction of cells embedded in the biofilm.

**Figure 12:**
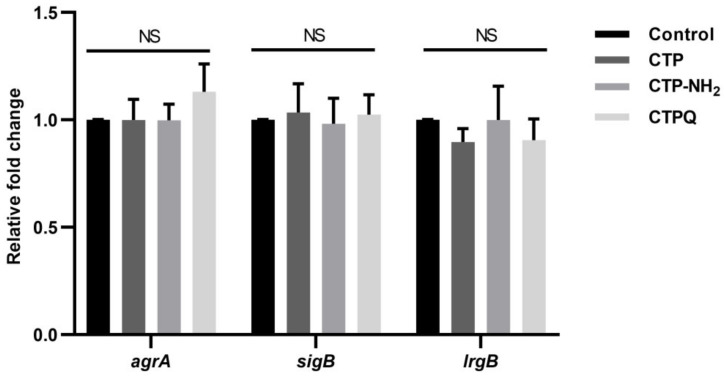
The qRT-PCR results of *S. aureus* ATCC 43300 genes related to biofilm treated with CTPs. NS: not significant (*p* > 0.05) (*n* = 3).

Finally, we conducted qRT-PCR assays to investigate whether CTPs could modulate the transcription levels of genes related to biofilm formation. There was no significant difference (*p* > 0.05) in the relative fold change of gene expression in the biofilms treated with CTPs at 2 × MIC compared to the control (Figure 12), which indicated that CTPs did not affect the gene transcription levels during biofilm formation.

### 2.8. Cell Viability and Anti-Adhesion Activity Tests

**Figure 13:**
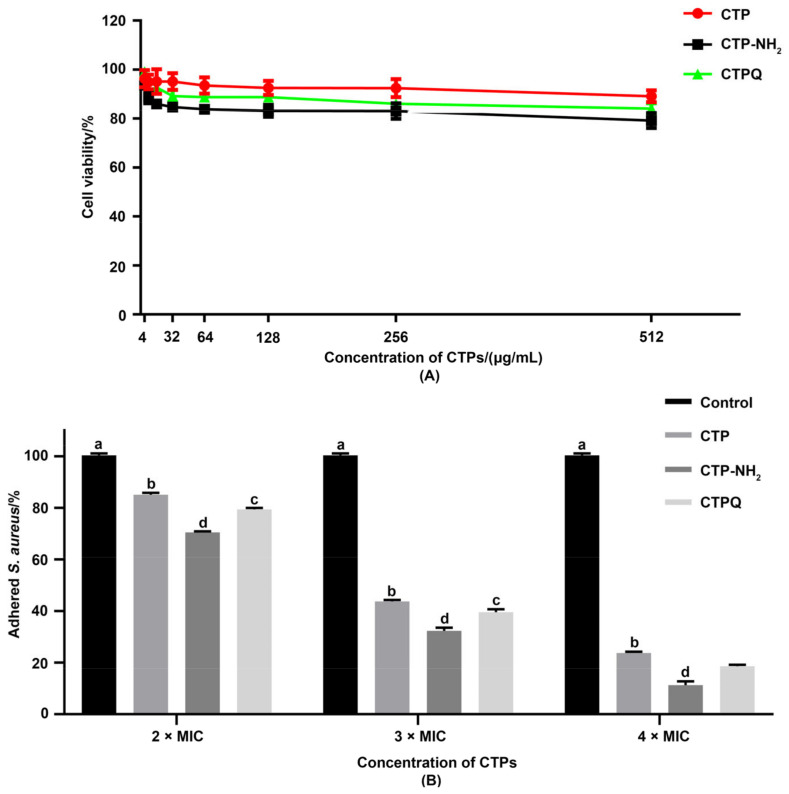
(**A**) Cell viability of intestinal porcine epithelial cell line (IPEC-J2) upon exposure to CTPs and (**B**) inhibitory effect of CTPs against the adherence of *S. aureus* ATCC 43300 to IPEC-J2 cells. ^a–d^: Different lowercase letters mean significantly different (*p* < 0.05) values were expressed as mean ± SEM (*n* = 3).

Adhesion is an essential step in biofilm formation. Therefore, we investigated the anti-adhesion effects of CTPs in vitro. First, the viability of the CTP-treated intestinal porcine epithelial cell line (IPEC-J2) was determined using a cell counting kit-8 (CCK-8) assay kit (Figure 13A). The IC20 values of CTPs against IPEC-J2 were all above 512 μg/mL, which is the maximum concentration used in this assay, proving that the CTPs had no significant cytotoxicity on the IPEC-J2 cells. Therefore, CTPs are available in the IPEC-J2 infection model in vitro.

To evaluate the anti-adhesion effects of CTPs against *S. aureus* ATCC 43300 on IPEC-J2 cells, CTPs were added to the medium and co-incubated with *S. aureus*. The solution containing the adhered bacteria was diluted and plated after infection for 3 h, followed by the calculation of the relative adherence rate (Figure 13B). Compared to the untreated group, co-incubation of *S. aureus* and CTPs at all of the tested concentrations significantly reduced (*p* < 0.05) the rate of bacterial adherence. Moreover, CTP-NH_2_ and CTPQ exhibited more potent anti-adhesion effects against *S. aureus* than CTP after incubation.

## 3. Discussion

The current study proves that CTPs exhibit effective antibacterial, antibiofilm, and anti-adhesion activities. This study contributes to our understanding of the possible antibacterial and antibiofilm mechanisms of CTPs, which may be developed as effective anti-MDR drug candidates.

Over the past few decades, the misuse of antibiotics has led to the emergence of DR and MDR bacteria [36]. Infectious diseases caused by these pathogens are now common [37], and there is an urgent need for new candidate drugs. Among the many antibiotic alternatives, AMPs have great potential as promising candidates. In addition, AMPs may impose less selective pressure on bacteria than traditional antibiotics, as they have been proven to act on multiple targets [38].

In our previous study, we designed and screened the α-helical cationic peptide, CTP, which could exert anti-inflammatory activity by neutralizing lipopolysaccharide (LPS) and competitively inhibiting the binding of LPS to TLR4/MD2 complex receptors on the cell membrane surface [28]. In addition, modification of the CTP peptide by C-terminal amidation (CTP-NH_2_) was able to enhance its anti-inflammatory activity and reduce its cytotoxicity to RAW 264.7 cells [28]. However, amidation of the C-terminus is difficult to achieve directly in a heterologous expression. Some reports have shown that the addition of a residue with an amide group to the C-terminus of AMPs can significantly improve its antibacterial activity [29,30]. Therefore, we designated CTPQ as a CTP with a glutamine residue at the C-terminus. Importantly, the antibacterial and antibiofilm activities of these CTPs still needed to be evaluated.

Therefore, in the present study, one of our main objectives was to evaluate the antibacterial effect of CTPs against different strains, especially the standard MRSA strain *S. aureus* ATCC 43300. On basis of determining the MICs and MBCs against a wide range of bacteria, we found that CTPs had a broad-spectrum antibacterial activity against both gram-negative and gram-positive bacteria (Table 2). The antimicrobial activity of AMPs depends mainly on their physicochemical properties, including net charge number, hydrophobicity, and amphiphilicity [39]. Among the three peptides, CTP-NH_2_ showed the strongest antimicrobial activity, reflected by the lowest MICs and MBCs on most of the tested strains, which was consistent with Zhang’s report [28] that the hydrophobicity of CTP-NH_2_ was stronger than that of the original peptide caused by the C-terminal amidation, increasing its antimicrobial activity [40,41]. Moreover, the MICs of CTP and CTPQ were identical for most of the tested strains, suggesting that they had almost the same antibacterial activity. On basis of predicting the physicochemical properties of the peptides online, it was also found that CTP and CTPQ exhibited certain differences in their physicochemical properties (Table 2, Figure 1). Therefore, the differences in antibacterial properties among the three peptides analyzed in this study may also likely be related to their essential physicochemical properties. Our results indicated that the addition of glutamine at the C-terminal of CTP could improve the antibacterial effects of CTP against *P. aeruginosa* ATCC 27853 and *P. aeruginosa* CGMCC 1.10712.

The ability of AMPs to directly damage bacterial cell membranes and rapidly kill bacteria has been reported [42]. Cationic peptides are positively charged and can selectively bind to the negatively charged components of bacterial cell membranes, such as the LPS of gram-negative bacteria and lipoteichoic acid of gram-positive strains [43]. Then, the peptides tend to insert into the cell membrane mediated by hydrophobic forces, followed by aggregating with each other and forming pores on the membrane, causing the outflow of the cell contents and eventually leading to lysis and death of the bacteria [44]. We found by using prediction tools that all of the CTPs were cationic peptides (Table 1). Therefore, we further explored whether the bactericidal mechanisms of these peptides were mediated through the disruption of the bacterial cell membrane. In this study, all of the CTPs were able to increase the uptake of PI at the MIC and the MBC, which demonstrated that the cell membrane of *S. aureus* was disrupted by CTPs (Figure 6). Moreover, CTPs induced higher PI uptake at the MBC (2 × MIC) than the MIC, which may suggest that a higher concentration of CTPs could induce more severe damage to the cell membrane. Similar results were also observed in other studies [45,46], where cationic peptides increased the uptake of PI in microbial cells.

The SEM results showed that the cell membrane of *S. aureus* ATCC 43300 treated with CTPs at the MIC became rough and swollen, losing its original morphology (Figure 7). In addition, intracellular matrix leakage was observed as a result of membrane disruption, leading the cells to aggregate and stick together. Similarly, the broken cell membranes of CTP-treated *S. aureus* were also observed using TEM (Figure 8), which showed that the boundaries of both the cell walls and membrane were blurred. More importantly, cell membrane disruption in CTP-treated *S. aureus* induced further intracellular damage, such as DNA aggregation, ribosome cohesion, and cytoplasmic solidification, which eventually caused cell lysis. In summary, it was apparent that the main bactericidal mechanism of CTPs was to penetrate the cell membrane of *S. aureus* and further change the cell morphology and structure. Other studies also suggested that cationic α-helical peptide treatment could result in membrane atrophy, corrugation, and damage of *S. aureus* cells [47,48], which was consistent with our results.

Biofilm production can aggravate pathogenic infection [4]. Based on the significant antibacterial effects of CTPs at the MBC (64 μg/mL) on planktonic *S. aureus* (5 × 10^4^ CFU/mL), we further explored whether CTPs could also effectively inhibit biofilm formation by *S. aureus* (7.8 × 10^7^ CFU/mL). All of the CTPs exhibited significant antibiofilm activity and could effectively inhibit biofilm formation of most tested *S. aureus* strains at sublethal concentrations (1 × MIC), as detected using the crystal violet method (Figure 9). This was consistent with other reports, which suggest that membrane-penetrated peptides may also block the formation of pathogen biofilms [49,50]. C-terminal amidation of the peptides has been reported to enhance its antibiofilm activity [51]. Similarly, in this study, CTP-NH_2_ showed better antibiofilm activity than CTP and CTPQ (Figure 9). Interestingly, we found that CTPQ also showed a relatively higher antibiofilm activity than CTP against *S. aureus* ATCC 25923 and *S. aureus* CVCC 1882 (Figure 9D,E), which indicated that the addition of glutamine to the C-terminus of CTP could partially enhance its antibiofilm activity. Subsequently, by CLSM assay, we found that CTP-NH_2_ could inhibit the generation of biofilms by inhibiting bacterial proliferation and killing bacteria (Figure 11). Although CTP- and CTPQ-treated biofilm samples had relatively more visible cells, there was a higher proportion of dead cells than the control group (Figure 11). These results suggested that CTPs could effectively control the amount of viable bacteria during biofilm formation.

**Figure 14:**
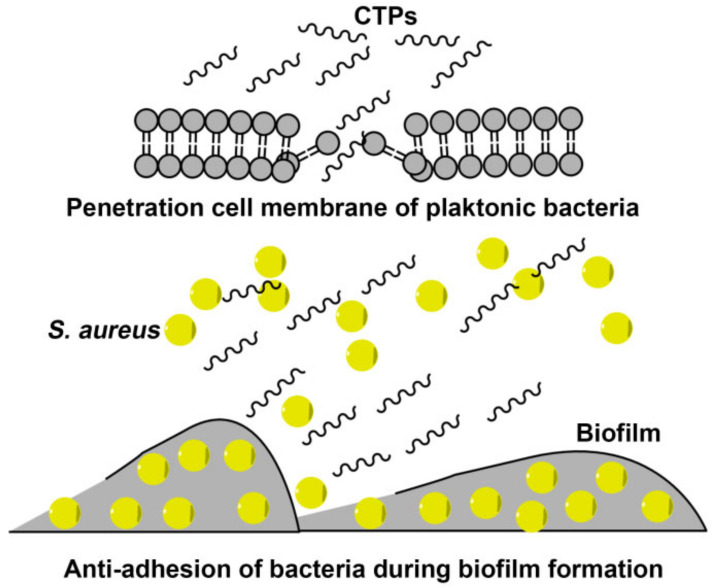
The hypothesized model of antibacterial and antibiofilm effects of CTPs against *S. aureus* ATCC 43300.

We simultaneously analyzed the transcriptional levels of biofilm-related genes in *S. aureus* ATCC 43300 by qRT-PCR. An important gene in the Agr QS system of *S. aureus*, *agrA*, can regulate the behavior of the entire population [49,52]. Furthermore, *sigB* expression down-regulates protease production, but promotes the expression of adhesion factors, which contributes to the initial formation of biofilms [53]. In addition, *lrgB* is a negative regulator of autolysis and can contribute to the inhibition of cell lysis and eDNA release [54]. In our study, the transcriptional levels of genes (*agrA*, *sigB*, and *lrgB*) in CTPs-treated biofilms were not significantly different, which might prove that the antibiofilm activity of CTPs mainly relied on membrane destruction, instead of the regulation of gene expression (Figure 12). However, these results may differ from Jiale’s report that AMP 1018 M could display both disruption of the cell membrane and modulation of biofilm-associated gene expression, which might be due to the target of 1018 M function being ppGpp, a stringent response signaling molecule that regulates the expression of many biofilm-formation-relevant genes [49]. As shown in Figure 14, we speculated on the mechanisms of antibacterial and antibiofilm effects of CTPs against *S. aureus* ATCC 43300 in the hypothesized model.

*S. aureus* is one of the most important pathogens, accounting for a variety of diseases, especially gastroenteritis [55,56,57]. Since the adhesion of pathogens to epithelial cells is an essential step for the pathogens to survive and colonize the gastrointestinal tract [10], there exists an urgent need for drugs that inhibit bacterial adhesion to the host [55]. In this study, we established an in vitro model of *S. aureus* ATCC 43300 infection using IPEC-J2 cells. First, our research showed that CTPs had low cytotoxicity against the IPEC-J2 cell line. In addition, we found that the adherence rate of *S. aureus* to IPEC-J2 cells was significantly decreased in a dose-dependent manner when CTPs and *S. aureus* ATCC 43300 were co-incubated, which was consistent with the results of Yu’s report [58] indicating that bovine lactoferrin peptide showed significant anti-adhesion effects against *S. aureus* in IPEC-J2 cells. Herein, our study indicated that CTPs possessed strong anti-adhesion effects against *S. aureus*, significantly weakening their adhesion ability to IPEC-J2 cells, along with minimal cytotoxicity.

## 4. Materials and Methods

### 4.1. Strains and Peptides

All of the strains (*S. aureus* ATCC 6385, *S. aureus* ATCC 43300, *S. aureus* CVCC 1882, *S. aureus* ATCC 25923, *S. castellani* CMCC 51592, *S. typhimurium* ATCC 14028, *S. pullorum* CVCC 519, *E. coli* ATCC K99, EHEC O157 H7, *P. aeruginosa* ATCC 27853, *P. aeruginosa* ATCC 9027, *P. aeruginosa* CGMCC 1.10712) used in this study were maintained in our laboratory. The peptides CTP and CTPQ were synthesized in the free C-terminal form, and CTP-NH_2_ was synthesized by C-terminal amidation. All of the peptides (purity ≥ 95%) were synthesized, purified, and tested (high-performance liquid chromatography (HPLC) and mass spectrometry) by GL Biochem Ltd. (Shanghai, China). The synthesized peptides were stored at −80 °C.

### 4.2. Physicochemical Properties of Peptides

The physicochemical properties of CTPs were predicted online in the database of antimicrobial activity and structure of peptides (DBAASPv3.0, https://www.dbaasp.org/ (accessed on 15 February 2021)). The helical wheels of peptides were predicted on the following website (https://www.bioinformatics.nl/cgi-bin/emboss/pepwheel (accessed on 12 February 2021)).

### 4.3. Determination of MICs and MBCs

The broth microdilution assay was used to determine the MICs and MBCs according to the Clinical and Laboratory Standards Institute (CLSI) guidelines [59], with minor modifications. Sterilized Mueller–Hinton broth (MHB) medium (180 μL) and antimicrobial peptides (20 μL) were added to sterile 96-well plates at final peptide concentrations of 0.5–512 μg/mL. The bacteria were cultured to the logarithmic growth stage, followed by centrifugation, washing, dilution, and resuspension. Then, 2 μL bacterial solution with a concentration of 5 × 10^6^ CFU/mL was added to the 96-well plates. The wells with only bacterial suspension or MHB medium were used as controls. The plates were incubated overnight at 37 °C in an incubator shaker at 200 rpm. The minimum peptide concentration at which bacterial growth was invisible in the 96-well plate was defined as the MIC of the CTPs.

The MBCs were tested after determining the MICs. A 20 μL aliquot of culture medium in the wells without visible bacterial growth was spread on MHB agar plates and cultured overnight at 37 °C to determine the MBC based on the absence of bacterial colony growth. Each trial was repeated three times.

### 4.4. Inhibition Zones Assay

Inhibition zones were assessed using the Oxford cup test [60]. The tested strains were cultured overnight and diluted to 10^7^ CFU/mL in MHB medium. The diluted bacterial solution (100 μL) was spread on MHB plates with several sterile Oxford cups containing 100 μL solutions of CTPs. All of the plates were incubated at 37 °C for 12 h, and the sizes of the growth inhibition zones were measured using Vernier calipers.

### 4.5. Time-Kill Curve Assay

*S. aureus* ATCC 43300 was inoculated into MHB medium and incubated overnight at 37 °C and 200 rpm. The culture medium was then incubated again in fresh MHB medium and diluted to 5 × 106 CFU/mL. The diluted bacterial solution (100 μL) was added to the shaking tubes along with 1 mL of CTPs (0, 1, and 2 × MIC) dissolved in sterile water and 9 mL MHB medium and mixed well. The tubes were then cultured at 37 °C and 200 rpm. During the cultivation, samples were collected at intervals of 3 h (0, 3, 6, and 9 h) and plated after dilution. The time-kill curves were measured using a spiral inoculator and a colony counter (Interscience, Cologne, Germany). The test was repeated three times.

### 4.6. Enumeration of Viable S. aureus

The assay was performed as reported by Shu et al. [61]. The preliminary growth and CTP treatment was the same as for the time-kill curves. After sampling at 6 h, all of the samples were diluted, and 3 μL of each diluted suspension was spotted on MHB plates and incubated at 37 °C for 12 h. The bactericidal effect of CTPs against *S. aureus* was evaluated based on an intuitive observation of the remaining colonies on the plates. The experiment was repeated three times.

### 4.7. CRA Plate Assay of S. aureus

Since biofilm formation is associated with slime production, we performed a CRA plate assay as previously described by Kannappan et al. [35] to determine the presence of slime. Sterile Congo red (0.8 g/L) was added to the mixed medium (15 g/L tryptone, 5 g/L peptone, 5 g/L NaCl, 36 g/L sucrose, and 20 g/L agar) at 55 °C. *S. aureus* cells treated with CTPs for 6 h and untreated *S. aureus* suspensions were spread on the CRA plates and incubated for 24 h.

### 4.8. Assessment of S. aureus Cell Membrane Integrity

The cell membrane integrity was assessed by analyzing the propidium iodide (PI) uptake. The *S. aureus* ATCC 43300 strain was cultured overnight in MHB medium and centrifuged at 10,000× *g* for 5 min at 25 °C. The harvested cell pellet was resuspended in a prepared PI-phosphate-buffered saline (PBS) solution (10 μM) and diluted to 5 × 10^6^ CFU/mL. Then, 180 μL of the suspension and 20 μL of the CTPs were added into a black 96-well plate and incubated at 37 °C for 160 min in the dark. The PI fluorescence intensity was measured every 20 min using a microplate reader, with an excitation wavelength of 535 nm and an emission wavelength of 617 nm [56].

### 4.9. SEM Assay of S. aureus

To investigate the bactericidal mechanism of CTPs, the cell surface of CTP-treated *S. aureus* was observed by SEM, as reported previously [62]. Briefly, 1 mL of 5 × 10^6^ CFU/mL *S. aureus*, 10 mL of CTP solution (0, 1 × MIC), and 90 mL MHB medium were mixed and incubated for 6 h at 37 °C and 200 rpm. The bacterial suspension was centrifuged at 7500 rpm, and 4 °C for 5 min, and washed thrice with sterile PBS. The cells were gently re-suspended in 1.5 mL of 4% glutaraldehyde fixing solution (Solarbio Life Sciences, Beijing, China) and fixed at 4 °C for 12 h. Then, the fixed cells were dehydrated in increasing concentrations of ethanol for 10 min each. Thereafter, the specimens were subjected to critical point drying and gold sputtering coating. Finally, the surface morphology of *S. aureus* ATCC 43300 was observed using cold field emission SEM (PP3010T, Quorum Technologies Ltd., Lewes, UK).

### 4.10. TEM Assay of S. aureus

For the TEM analysis, the samples were collected and fixed as described for the SEM assay. After gradient dehydration in ethanol solutions, the cells were soaked in a 1:1 mixture of acetone/embedding solution for 2 h and then in a pure embedding solution overnight. Finally, ultrathin sections were stained and observed using a TEM (Tecnai Spirit D1319, FEI, Brno, Czech Republic).

### 4.11. Antibiofilm Activity Assay

The test strains of *S. aureus* and *P. aeruginosa* were cultured overnight and diluted to 7.8 × 10^7^ CFU/mL. The bacterial suspension (90 μL) was added to 96-well plates containing 10 μL solutions of CTPs (1/2, 1, 2, and 4 × MIC). In the control wells, 10 μL sterile water was added instead of any antimicrobial peptide. The plates were incubated at 37 °C for 24 h. The supernatant was removed and the wells were washed twice gently with 150 μL/well PBS to remove planktonic bacteria and cell debris. The biofilm adhering to the bottom of the wells was stained with 125 μL/well crystal violet (0.1% *w/v*) and incubated for 10 min at 25 °C, followed by washing twice with 200 μL deionized water. The stained biofilm was dissolved in 200 μL/well ethanol for 10 min. Finally, 100 μL of the mixture was transferred to a clean 96-well plate, and the absorbance (Abs) at 600 nm was recorded using a microplate reader. The inhibition rates of biofilm formation were calculated using the following formula:

$$\%\;\mathrm{Biofilm}\;\mathrm{inhibition}\;\mathrm{rate}=\frac{\mathrm{Abs}\;\mathrm{of}\;\mathrm{control-Abs}\;\mathrm{of}\;\mathrm{sample}}{\mathrm{Abs}\;\mathrm{of}\;\mathrm{control}}\times 100$$

### 4.12. Optical Microscope Observation of Biofilm

The biofilms were prepared as described earlier but using cell slides in a 12-well plate as the biofilm carrier. The slides were then stained with crystal violet and gently washed. After desiccation, the biofilms on the slides were observed under an optical microscope at 400 × magnification.

### 4.13. CLSM Assay of Biofilm

Biofilms of *S. aureus* ATCC 43300 were prepared in CLSM Petri dishes. The planktonic bacteria were then discarded, and the biofilm was washed twice with sterile saline solution. A mixed SYTO 9/PI dye (200 μL, Invitrogen, Waltham, MA, USA) was gently added to the center of the Petri dish and incubated at 25 °C for 20–30 min in the dark. The surplus dye was then gently washed away twice with sterile saline. Thereafter, the Petri dish was covered with 1.5 mL sterile saline, and the biofilm was observed using a 40 × oil lens on CLSM (A1HD25, Nikon, Minato, Japan). Z-series images collected at 1.0 μm intervals were used to construct 3D images of the biofilm using the NIS-Elements Viewer 5.21 software.

### 4.14. Biofilm-Related Gene Expression Assays

The effects of CTPs on the transcriptional levels of genes related to biofilm formation, including accessory gene regulator protein A (*agrA*), antiholin-like protein B (*lrgB*), and RNA polymerase sigma factor B (*sigB*), were analyzed by a quantitative real-time polymerase chain reaction (qRT-PCR). Biofilms were prepared by the same method as described above. The cells in the biofilm were carefully collected and lysed with 250 U lysostaphin enzyme (Shanghai Hi-Tech Joint Biotechnology, Shanghai, China) for 45 min at 37 °C. The total RNA was extracted from *S. aureus* biofilms using the UNIQ-10 Column Trizol Total RNA Isolation Kit (Sangon Biotech, Shanghai, China) following the manufacturer’s protocol. A cDNA synthesis was performed using HiScript^®^ II Q Select RT SuperMix from the qRT-PCR kit (Novozymes, Beijing, China). A qRT-PCR was performed using a fluorescent quantitative PCR system (Light Cycler^®^ 96, Roche, Basel, Switzerland) with the SYBR^®^ Premix Ex Taq TM kit (Takara, Beijing, China). Table 3 lists the primers used in this experiment.

### 4.15. Cell Culture

The intestinal porcine epithelial cell (IPEC-J2) was bought from the American Type Culture Collection (ATCC) (Manassas, VA, USA) and maintained in our lab. The cell recovery process was conducted as follows: the IPEC-J2 cells stored in the liquid nitrogen were quickly incubated at 37 °C in a water bath heater for 2 min with continuous shaking; after the cell fluid inside the tube was completely thawed, the cell suspension was transferred to a centrifuge tube filled with 5 mL complete medium containing Dulbecco’s modified Eagle’s medium (DMEM) (Gibco, Foster, CA, USA), 10% (*v/v*) fetal bovine serum (FBS) (Gibco), and 1% (*v/v*) penicillin/streptomycin (HyClone, Logan, UT, USA); then, the cell suspension was centrifuged at 25 °C, 1000 rpm/min for 4 min, followed by discarding the supernatant and adding 1 mL complete medium to the cell precipitate; the cell suspensions were then mixed well and inoculated into the cell culture bottles and incubated at 37 °C in a cell incubator (5% CO_2_) until most of the cells adhered to the bottle; after the cell medium was replaced by fresh complete medium, the culture was continued until the cells covered 80–90% of the culture bottle.

Subsequently, cell passage was carried out as follows: after the culture medium was discarded, the adhered cells were gently washed by sterile PBS to remove the serum; then 2 mL 0.25% trypsin (HyClone, Logan, UT, USA) was added into the bottle and incubated in a cell incubator for 2–4 min to digest the adhered cells. All of the cell-digested suspension was mixed with an equal volume of the complete medium, and was then centrifuged at 25 °C, 1000 rpm/min for 4 min. After the supernatant was discarded, the cell precipitate was resuspended with 5 mL fresh complete medium in a new cell culture bottle, incubated at 37 °C in a cell incubator (5% CO_2_). After two passages, the cells were ready for the next tests.

### 4.16. Cell Viability Assay

The viability of the CTP-treated IPEC-J2 cells was determined using a cell counting kit-8 (CCK-8) assay kit (Solarbio). The IPEC-J2 cells (2 × 10^4^ cells/mL) were cultured overnight in 100 μL DMEM in 96-well plates. Fresh medium (100 μL) containing CTPs at different final concentrations (4, 8, 16, 32, 64, 128, 256, and 512 μg/mL) was added, followed by incubation for another 24 h. The medium was replaced with 100 μL fresh DMEM and 10 μL CCK-8 solution. After incubation at 37 °C for 2–4 h, the absorbance of the plates at 450 nm was determined using a microplate reader. The cell viability was calculated as follows:

$$\%\;\mathrm{Cell}\;\mathrm{viability}=\frac{\mathrm{Abs}\;\mathrm{of}\;\mathrm{samples}}{\mathrm{Abs}\;\mathrm{of}\;\mathrm{control}}\times 100$$

### 4.17. Bacterial Adherence Assays

Briefly, IPEC-J2 cells (1 mL, at a density of 2 × 10^5^ cells/mL in DMEM) were added to 24-well plates and cultured in a 37 °C incubator with 5% CO_2_ for 24 h. The supernatant waste medium was discarded and the cells were washed with PBS. Then, 1 mL of cell culture medium and *S. aureus* ATCC 43300 (1 × 10^6^ CFU/mL) suspension pretreated with CTPs were added to the wells. After a 3 h infection, the cells were carefully washed twice with PBS to remove the unattached bacterial suspension. Thereafter, 100 µL of 0.1% Triton X-100 (Solarbio) was added to the wells for 10 min, and 900 µL of MHB medium was added to dilute the bacteria. Finally, the bacterial cultures were spread on MHB plates for 12 h and the number of colonies formed was determined using a bacterial colony counter. Each adherence assay was repeated three times. The bacterial adherence rate was calculated as follows:

$$\%\;\mathrm{Bacterial}\;\mathrm{adherence}\;\mathrm{rate}=\frac{\mathrm{CFU}/\mathrm{mL}\;\mathrm{of}\;\mathrm{samples}}{\mathrm{CFU}/\mathrm{mL}\;\mathrm{of}\;\mathrm{control}}\times 100$$

### 4.18. Statistics

The data from all of the above tests are expressed as the mean ± SEM of at least three independent experiments. The data were first tested for normality and homogeneity of variance using SPSS version 19.0. Then, the data were subjected to one-way analysis of variance (ANOVA) with the general linear model, and the significant differences in all of the figures were tested by multiple comparisons. The statistical significance was set at *p* < 0.05.

## 5. Conclusions

Overall, it was found that CTPs had strong antibacterial activities against all of the tested gram-negative and gram-positive bacteria, which indicated that they might cause broad-spectrum antibacterial effects. The results of this study indicate that the bactericidal mechanism of CTPs originates from their ability to disrupt the cell membrane of pathogens. Furthermore, our results from antibiofilm assays showed that CTPs could significantly inhibit biofilm formation by *S. aureus* ATCC 43300 by killing the cells in the biofilm. Finally, we established an *S. aureus* infection model with IPEC-J2 cells, which indicated that CTPs possessed strong anti-adhesion effects against *S. aureus*, significantly suppressing its adhesion to IPEC-J2 cells, along with low cytotoxicity. These results provide the possible mechanisms of the novel multifunctional peptide CTPs and may contribute to the development of promising antimicrobial drug candidates for the treatment of *S. aureus* infections. Although this study has proven that CTPs have effective antibacterial, antibiofilm, and anti-adhesion activities in vitro, the corresponding functions of CTPs still need to be explored by animal models In vivo in future studies. The findings provided in the current study could help us to understand the possible functional mechanisms of the novel bioactive peptide CTPs and might contribute to the development of a promising candidate for the treatment of an infection caused by MRSA.

## Figures and Tables

**Table 1:** Sequence and physicochemical properties of CTPs.

Name of Peptide	Normalized Hydrophobic Moment	Normalized Hydrophobicity	Net Charge	Amphiphilicity Index
CTP	1.11	0.34	8.00	1.75
CTPQ	1.07	0.40	8.00	1.72

Note: CTP: cathelicidin 2 (CATH2) and thymopentin (TP5); CTPQ: addition of glutamine at the C-terminal of cathelicidin 2 (CATH2) and thymopentin (TP5).

**Table 2:** MICs and MBCs of CTPs on different strains.

Strains	Peptides	MICs (μg/mL)	MBCs (μg/mL)
*S. aureus* ATCC 6385	CTP	16	32
	CTPQ	16	32
	CTP-NH_2_	8	16
*S. aureus* ATCC 43300	CTP	32	64
	CTPQ	32	64
	CTP-NH_2_	32	64
*S. aureus* CVCC 1882	CTP	4	4
	CTPQ	4	4
	CTP-NH_2_	2	8
*S. aureus* ATCC 25923	CTP	64	512
	CTPQ	64	512
	CTP-NH_2_	32	512
*S. castellani* CMCC 51592	CTP	32	128
	CTPQ	32	128
	CTP-NH_2_	32	64
*S. typhimurium* ATCC 14028	CTP	32	128
	CTPQ	32	512
	CTP-NH_2_	32	64
*S. pullorum* CVCC 519	CTP	8	64
	CTPQ	8	64
	CTP-NH_2_	8	32
*E. coli* ATCC K99	CTP	32	256
	CTPQ	32	128
	CTP-NH_2_	16	64
EHEC O157 H7	CTP	16	256
	CTPQ	16	128
	CTP-NH_2_	8	128
*P. aeruginosa* ATCC 27853	CTP	64	128
	CTPQ	32	64
	CTP-NH_2_	16	64
*P. aeruginosa* ATCC 9027	CTP	64	256
	CTPQ	64	128
	CTP-NH_2_	32	128
*P. aeruginosa* CGMCC 1.10712	CTP	64	128
	CTPQ	32	64
	CTP-NH_2_	8	64

Note: CTP: cathelicidin 2 (CATH2) and thymopentin (TP5); CTP-NH_2_: amidation-modified C-terminal of cathelicidin 2 (CATH2) and thymopentin (TP5); CTPQ: addition of glutamine at the C-terminal of cathelicidin 2 (CATH2) and thymopentin (TP5).

**Table 3:** Primer sequences required for qRT-PCR genes.

Gene Name	Primer Sequence	ATCC Genome Locus_Tag ^1^
*agrA*	F: 5′-CTGATAATCCTTATGAGGTGCTTGA-3′	KNNFDEDG_02655
	R: 5′-CGTAAGTTCACTGTGACTCGTAACG-3′	
*lrgB*	F: 5′-ACTACAGCGATTGCGTTACCA-3′	KNNFDEDG_01697
	R: 5′-CTTGCCATTGATTCTTCTACAGGT-3′	
*sigB*	F: 5′-TTGACCATTCCATTGAAGCTG-3′	KNNFDEDG_02624
	R: 5′-AACCGATACGCTCACCTGTC-3′	

Note: ^1^: All of the cDNA fragments were obtained from the genomic data of *S. aureus* ATCC^®^ 43300 in the ATCC Genome Portal (https://genomes.atcc.org/ (accessed on 5 June 2021)).
